# Potential scrapie-associated polymorphisms of the prion protein gene (*PRNP*) in Korean native black goats

**DOI:** 10.1038/s41598-019-51621-y

**Published:** 2019-10-25

**Authors:** Seon-Kwan Kim, Yong-Chan Kim, Sae-Young Won, Byung-Hoon Jeong

**Affiliations:** 1 Korea Zoonosis Research Institute, Chonbuk National University, Iksan, 54531 Republic of Korea; 2 Department of Bioactive Material Sciences and Institute for Molecular Biology and Genetics, Chonbuk National University, Jeonju, 54896 Republic of Korea

**Keywords:** Genetic markers, Haplotypes

## Abstract

Small ruminants, including sheep and goats are natural hosts of scrapie, and the progression of scrapie pathogenesis is strongly influenced by polymorphisms in the prion protein gene (*PRNP*). Although Korean native goats have been consumed as meat and health food, the evaluation of the susceptibility to scrapie in these goats has not been performed thus far. Therefore, we investigated the genotype and allele frequencies of *PRNP* polymorphisms in 211 Korean native goats and compared them with those in scrapie-affected animals from previous studies. We found a total of 12 single nucleotide polymorphisms (SNPs) including 10 nonsynonymous and 2 synonymous SNPs in Korean native goats. Significant differences in allele frequencies of *PRNP* codons 143 and 146 were found between scrapie-affected goats and Korean native goats (p < 0.01). By contrast, in *PRNP* codons 168, 211 and 222, there were no significant differences in the genotype and allele frequencies between scrapie-affected animals and Korean native goats. To evaluate structural changes caused by nonsynonymous SNPs, PolyPhen-2, PROVEAN and AMYCO analyses were performed. PolyPhen-2 predicted “possibly damaging” for W102G and R154H, “probably damaging” for G127S. AMYCO predicted relatively low for amyloid propensity of prion protein in Korean native black goats. This is the first study to evaluate the scrapie sensitivity and the first *in silico* evaluation of nonsynonymous SNPs in Korean native black goats.

## Introduction

Small ruminants, including sheep and goat, are natural hosts of scrapie belonging to the group of transmissible spongiform encephalopathies (TSEs), which also includes bovine spongiform encephalopathy (BSE) in cattle, chronic wasting disease (CWD) in deer and elk, and kuru, fatal familial insomnia (FFI), Gerstmann-Sträussler-Scheinker syndrome (GSS) and Creutzfeldt-Jakob disease (CJD) in humans^1–7^. TSEs are characterized by neurodegenerative symptoms in brain tissue and are attributed to the conformational change from the normal prion protein (PrP^C^) to the deleterious isoform of prion protein (PrP^Sc^), which entails distributional changes in secondary structure of PrP^C^ ^8^.

Prion protein gene (*PRNP*) polymorphisms have a critical effect on prion disease susceptibility among a wide range of hosts^9,10^. In our previous studies, *PRNP* polymorphisms in Korean people, chickens, horses and cattle have been reported, and evaluation for susceptibility to prion diseases was performed^11–19^. In addition, several polymorphisms in paralogs of the *PRNP* gene have been reported in cattle and goats^20–23^. In humans, distributions of the single nucleotide polymorphisms (SNPs) at codons 129 and 219 of the *PRNP* gene are correlated with the susceptibility to sporadic and variant CJD^11^. For example, M129V and E219K heterozygotes are protective against the development of sporadic CJD. In addition, all variant CJD patients were 129MM homozygous^24^. In cattle, insertion/deletion polymorphisms in the regulatory region of the *PRNP* gene may influence the expression level of PrP^C^, leading to different incubation periods and an increase in BSE susceptibility^25,26^. According to recent studies, 23-bp insertion/deletion polymorphisms in the promoter region and 12-bp insertion/deletion polymorphisms in the promoter region of intron 1 of the *PRNP* gene are associated with BSE susceptibility^4,12,27^.

In sheep, several nonsynonymous SNPs of the *PRNP* gene have been identified at codons M112T, A136V, M137T, S138N, L141F, R151C, R154H, Q171R/H, N176K, and R211Q^5,28–30^. Among these alleles, heterozygosity at codons R154H and Q171R has been shown to have a protective effect against the development of classical scrapie^5,28–30^. Recently, several studies have shown that the ARR allele at codons 136, 154 and 171 of the *PRNP* gene is associated with a highly protective effect against natural or experimental infection with classical scrapie and BSE, while the VRQ and ARQ alleles of the *PRNP* gene are susceptible to classical scrapie and BSE in sheep^3,31–33^. According to previous studies, a number of *PRNP* polymorphisms such as V21A, L23P, G37V, G49S, W102G, T110N, T110P, G127S, L133Q, M137I, I142M, H143R, N146S, N146D, R151H, R154H, P168Q, R211Q, I218L, Q220H, Q222K and S240P have been identified in goats^3,9,34–40^. Among these alleles, the heterozygosity at codons I142M, H143R, N146S, R211Q and Q222K confers decreased susceptibility to scrapie development in goats.

Korean native black goats are the only Korean indigenous breed that has been farmed for over 2,000 years. According to the Statistics Korea (http://kostat.go.kr/portal/korea/index.action) 2015 survey, Korean native black goats are known as the only breed raised in Korea. In addition, 300,000 heads of Korean native black goats were raised in 9,400 farm houses and were consumed as meat and health food. To date, scrapie in goat has not been reported in Korea. In addition, the estimation of the susceptibility to scrapie has not been investigated in Korean native black goats thus far.

The purpose of this study was to evaluate the degree of potential scrapie susceptibility in Korean native black goats. Thus, we investigated the genotype and allele frequencies of *PRNP* polymorphisms in 211 Korean native black goats and compared them with those of scrapie-affected animals in previous studies. In addition, we investigated linkage disequilibrium (LD) and analyzed haplotypes of the *PRNP* polymorphisms. Furthermore, we also evaluated the biological impact, such as the protein structure and functions of nonsynonymous SNPs, using PolyPhen-2, PROVEAN and AMYCO analyses.

## Results

### Investigation of genetic characteristics of the *PRNP* gene in 211 Korean native black goats

We performed automatic direct sequencing at the open reading frame (ORF) of the *PRNP* gene in 211 Korean native black goats. The sequenced ORF in the Korean native black goats was 771 bp in length and homologous with the *PRNP* gene of *Capra hircus* registered in the GenBank website (Gene ID: EU870890.1). We found a total of 12 SNPs, including 10 nonsynonymous SNPs. The genotype and allele frequencies of the caprine *PRNP* gene are shown in Table 1. Previous reported 3 nonsynonymous SNPs, c.426A > G (I142M), c.503C > A (P168Q), and c.664C > A (Q222K), were not found in Korean native black goats. Except for c.632G > A (R211Q), all genotype frequencies of SNPs were in Hardy-Weinberg Equilibrium (HWE) proportions.

**Table 1:** Genotype and allele frequencies of twelve *PRNP* polymorphisms in Korean native black goats.

	Genotype frequency, n (%)			Allele frequency, n (%)		*HWE
c.126G > A	GG	GA	AA	G	A	
42P	114 (54.03)	78 (36.97)	19 (9.00)	306 (72.51)	116 (27.49)	0.291
c.302A > G	AA	AG	GG	A	G	
Q101R	209 (99.05)	2 (0.95)	0 (0.00)	420 (99.53)	2 (0.47)	0.945
c.304T > G	TT	TG	GG	T	G	
W102G	197 (93.36)	14 (6.64)	0 (0.00)	408 (96.68)	14 (3.32)	0.618
c.379G > A	GG	GA	AA	G	A	
G127S	203 (96.21)	8 (3.79)	0 (0.00)	414 (98.10)	8 (1.90)	0.779
c.414T > C	TT	TC	CC	T	C	
138S	121 (57.35)	74 (35.07)	16 (7.58)	316 (74.88)	106 (25.12)	0.325
c.426A > G	AA	AG	GG	A	G	
I142M	211 (100)	0 (0.00)	0 (0.00)	422 (100)	0 (0.00)	
c.428A > G	AA	AG	GG	A	G	
H143R	103 (48.82)	87 (41.23)	21 (9.95)	293 (69.43)	129 (30.57)	0.6773
c.437A > G	AA	AG	GG	A	G	
N146S	193 (91.47)	17 (8.06)	1 (0.47)	403 (95.50)	19 (4.50)	0.360
c.461G > A	GG	GA	AA	G	A	
R154H	210 (99.53)	1 (0.47)	0 (0.00)	421 (99.76)	1 (0.24)	0.973
c.503C > A	CC	CA	AA	C	A	
P168Q	211	0	0	422	0	
c.512A > G	AA	AG	GG	A	G	
Q171R	210 (99.53)	1 (0.47)	0 (0.00)	421 (99.76)	1 (0.24)	0.973
c.632G > A	GG	GA	AA	G	A	
R211Q	207 (98.10)	3 (1.42)	1 (0.47)	417 (98.82)	5 (1.18)	<0.01
c.652A > C	AA	AC	CC	A	C	
I218L	208 (98.58)	3 (1.42)	0 (0.00)	419 (99.29)	3 (0.71)	0.917
c.664C > A	CC	CA	AA	C	A	
Q222K	211 (100)	0 (0.00)	0 (0.00)	422 (100)	0 (0.00)	
c.718C > T	CC	CT	TT	C	T	
P240S	135 (63.98)	67 (31.75)	9 (4.27)	337 (79.86)	85 (20.14)	0.851

^*^HWE: Hardy–Weinberg equilibrium.

We also investigated the LD among the 12 SNPs of the caprine *PRNP* gene by analyzing Lewontin’s D’ (|D’|) values (Table 2). The SNP c.379G > A showed low LD with 3 other SNPs (c.126G > A (0.511), c.414T > C (0.548), and c. 461G > A (0.492). In addition, c.718C > T showed low LD with c.461G > A (0.375). The remaining SNPs showed strong LD with a score range of 0.9–1.0.

**Table 2:** Linkage Disequilibrium (LD) of twelve *PRNP* polymorphisms in Korean native black goats.

	c.126G > A 42P	c.302A > G Q101R	c.304T > G W102G	c.379G > A G127S	c.414T > C 138S	c.428A > G H143R	c.437A > G N146S	c.461G > A R154H	c.512A > G Q171R	c.632G > A R211Q	c.652A > C I218L	c.718C > T P240S
c.126G > A 42 P	—	1.0	1.0	0.511	1.0	1.0	1.0	1.0	1.0	1.0	1.0	0.983
c.302A > G Q101R	—	—	1.0	1.0	1.0	1.0	1.0	1.0	1.0	1.0	1.0	1.0
c.304T > G W102G	—	—	—	1.0	1.0	1.0	1.0	1.0	1.0	1.0	1.0	1.0
c.379G > A G127S	—	—	—	—	0.548	1.0	1.0	0.492	1.0	1.0	1.0	1.0
c.414T > C 138S	—	—	—	—	—	1.0	1.0	1.0	1.0	1.0	1.0	0.984
c.428A > G H143R	—	—	—	—	—	—	1.0	1.0	1.0	1.0	1.0	0.923
c.437A > G N146S	—	—	—	—	—	—	—	1.0	1.0	1.0	1.0	1.0
c.461G > A R154H	—	—	—	—	—	—	—	—	1.0	1.0	1.0	0.375
c.512A > G Q171R	—	—	—	—	—	—	—	—	—	1.0	1.0	1.0
c.632G > A R211Q	—	—	—	—	—	—	—	—	—	—	1.0	1.0
c.652A > C I218L	—	—	—	—	—	—	—	—	—	—	—	1.0
c.718C > T P240S	—	—	—	—	—	—	—	—	—	—	—	—

Next, we examined the haplotype frequency of these 10 *PRNP* nonsynonymous SNPs. As shown in Table 3, 8 major haplotypes were identified. Among the 8 haplotypes, the haplotype QWGHNRQRIP had the highest frequency (36.3%), followed by QWGRNRQRIP (30.3%) and QWGHNRQRIS (14.6%).

**Table 3:** Haplotype frequencies of 10 nonsynonyomous single nucleotide polymorphisms of *PRNP* gene in Korean native black goats.

Haplotypes	c.302A > G	c.304T > G	c.379G > A	c.428A > G	c.437A > G	c.461G > A	c.512A > G	c.632G > A	c.652A > C	c.718C > T	N = 422
	Q101R	W102G	G127S	H143R	N146S	R154H	Q171R	R211Q	I218L	P240S	
Haplotype 1	Q	W	G	H	N	R	Q	R	I	P	153 (0.363)
Haplotype 2	Q	W	G	R	N	R	Q	R	I	P	128 (0.303)
Haplotype 3	Q	W	G	H	N	R	Q	R	I	S	62 (0.146)
Haplotype 4	Q	W	G	H	S	R	Q	R	I	P	19 (0.045)
Haplotype 5	Q	W	G	H	N	R	Q	R	I	P	16 (0.039)
Haplotype 6	Q	G	G	H	N	R	Q	R	I	S	14 (0.033)
Haplotype 7	Q	W	G	H	N	R	Q	R	I	P	15 (0.036)
Haplotype 8	Q	W	S	H	N	R	Q	R	I	P	5 (0.012)
Others^a^											10 (0.023)

Others^a^ contain rare haplotype with frequency < 0.01.

### Evaluation of potential scrapie susceptibility in Korean native black goats

**Figure 1:**
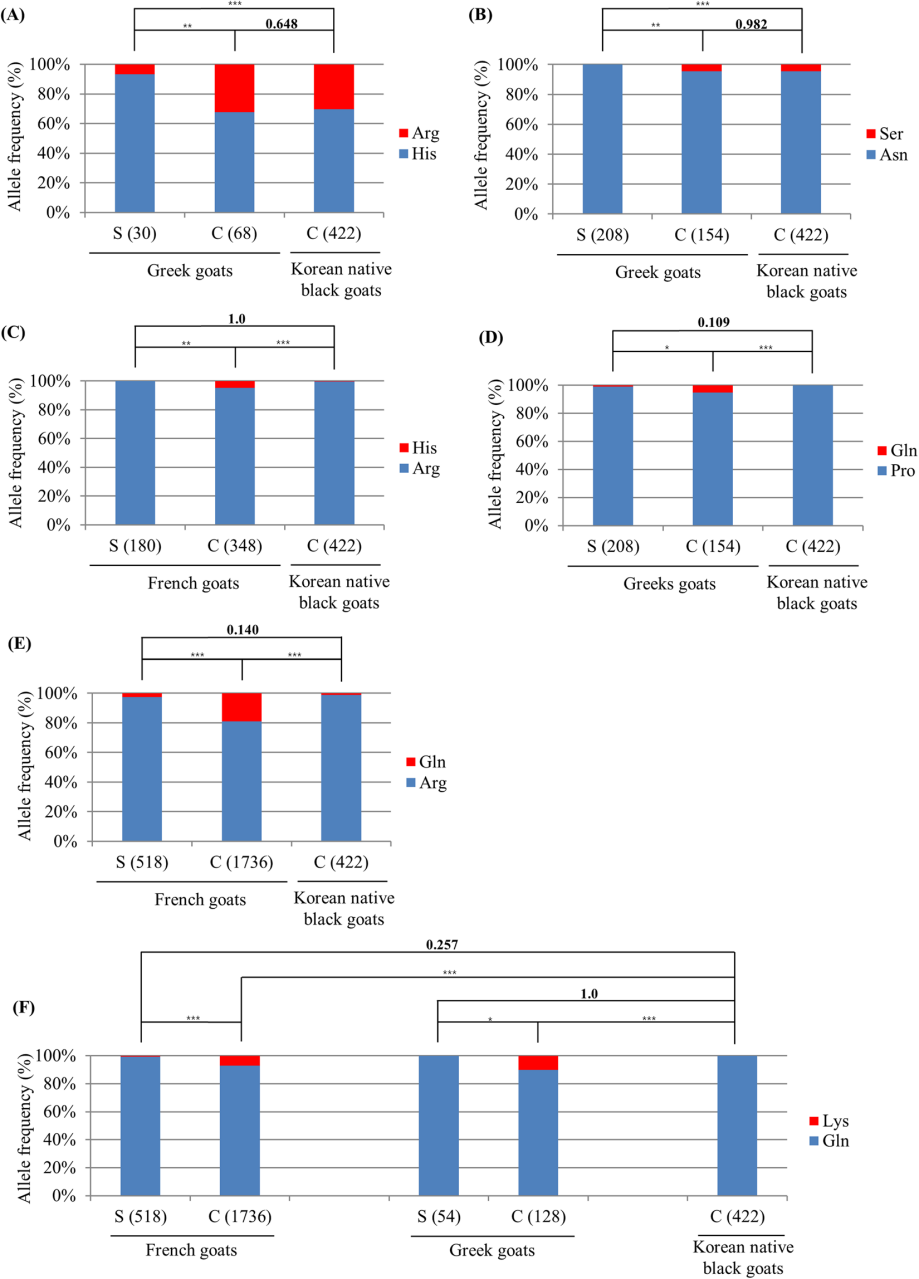
Comparisons of the allele frequencies of *PRNP* codons 143, 146, 168, 211, and 222 in Greek, French and Korean goats. (**A**) Comparisons of the allele frequency of the *PRNP* codon 143 between Greek goats and Korean native black goats^3^. (**B**) Comparisons of the allele frequency of *PRNP* codon 146 between Greek goats and Korean native black goats^50^. **(C)** Comparisons of the allele frequency of the *PRNP* codon 154 between French goats and Korean native black goats^58^. (**D**) Comparisons of the allele frequency of *PRNP* codon 168 between Greek goats and Korean native black goats^50^. (**E**) Comparisons of the allele frequency of *PRNP* codon 211 between French goats and Korean native black goats^33^. (**F**) Comparisons of the allele frequency of *PRNP* codon 222 among French goats, Greek goats and Korean native black goats^33,41^. Differences in allele distributions were calculated by chi-squired (χ^2^) tests and Fisher’s exact test. S: scrapie-affected goats, C: healthy goats.

To evaluate potential scrapie susceptibility in Korean native black goats, we compared the genetic distribution of scrapie-associated SNPs (R143H, N146S, R154H, P168Q, R211Q and Q222K) between Korean native black goats and scrapie-affected goats in other countries. These 6 scrapie-associated SNPs of the caprine *PRNP* gene, which were reported previously, were selected to evaluate potential scrapie susceptibility in Korean native black goats^3,6,33,34,37–39,41,42^. In *PRNP* codons 143 and 146, there was a significant difference in allele frequencies between scrapie-affected Greek goats and healthy Greek goats (p < 0.01). In addition, a significant difference in allele frequencies of *PRNP* codons 143 and 146 was found between scrapie-affected Greek goats and Korean native black goats (p < 0.01) (Fig. 1A,B). The allele frequencies at *PRNP* codon 154 showed significant differences between scrapie-affected French goats and healthy French goats (p = 0.0011). Interestingly, the allele frequencies of this SNP were not significantly different between scrapie-affected French goats and Korean native black goats (p = 1.0) (Fig. 1C). The allele frequencies at *PRNP* codon 168 showed significant differences between scrapie-affected Greek goats and healthy Greek goats (p < 0.05). Interestingly, the allele frequencies of this SNP were not significantly different between scrapie-affected Greek goats and Korean native black goats (p = 0.109) (Fig. 1D). In French goats, a significant difference in allele frequencies at codon 211 of the *PRNP* gene was found between scrapie-affected goats and healthy goats (p < 0.001). There was no significant difference in the allele frequencies between French scrapie-affected goats and Korean native black goats (Fig. 1E). In *PRNP* codon 222 of French and Greek goats, significant differences in allele frequencies were detected between scrapie-affected goats and healthy goats. In addition, a significant difference in allele frequencies at this codon of the *PRNP* gene was found between French and Greek healthy goats and Korean native black goats (p < 0.001) (Fig. 1F).

### Comparison of genetic distributions of *PRNP* codons 136, 154 and 171 in Korean native black goats and goats in other countries

We investigated the caprine *PRNP* haplotypes of codons 136, 154 and 171 in Korean native goats. In addition, the *PRNP* haplotypes were compared with those previously reported in 4 countries, namely, Pakistan, China, Japan and France^33,43–45^. In all countries, the ARQ haplotype was distributed over 97% of goats. In Pakistani goats, Chinese goats and Japanese goats, the frequency of the detected AHQ haplotype was less than 1%. Notably, ARR was found in over 1% of Japanese goats, and AHQ was found in 2.39% of French goats. In French goats, a significant difference was found in the allele distribution of the AHQ haplotype between scrapie-affected goats and healthy goats (p < 0.001)^33^. In addition, more than 95% of the ARQ/ARQ genotype was distributed in all countries, and significant differences in the distribution of the ARQ/AHQ genotype in French goats were found between scrapie-affected goats and healthy goats. (p < 0.001). There were few ARQ/ARR genotypes in Korea (0.47%) and Japan (1.34%), and the ARR/ARR genotype was found only in Japan (0.67%) (Table 4).

**Table 4:** Distributions of haplotype and genotype frequencies at *PRNP* codons 136, 154 and 171 between scrapie affected goats and healthy goats.

References	Pakistani goats	Chinese goats	Japanese goats	French goats		*p*-value	Korean native black goats
	^43^	^44^	^45^	^33^			In this study
	Healthy	Healthy	Healthy	Scrapie	Healthy		Healthy
**Haplotype**							
ARQ	143 (99.31)	665 (99.85)	588 (98.33)	1036 (100)	3389 (97.61)	0.629	420 (99.53)
ARR	0 (0.00)	0 (0.00)	8 (1.34)	0 (0.00)	0 (0.00)		1 (0.24)
AHQ	1 (0.69)	1 (0.15)	2 (0.33)	0 (0.00)	83 (2.39)	<0.001	1 (0.24)
**Genotype**							
ARQ/ARQ	71 (98.61)	332 (99.70)	292 (97.66)	518 (100)	1653 (95.22)		209 (99.05)
ARQ/AHQ	1 (1.39)	1 (0.30)	0 (0.00)	0 (0.00)	83 (4.78)	<0.001	1 (0.47)
ARQ/ARR	0 (0.00)	0 (0.00)	4 (1.34)	0 (0.00)	0 (0.00)		1 (0.47)
ARR/ARR	0 (0.00)	0 (0.00)	2 (0.67)	0 (0.00)	0 (0.00)		0 (0.00)
AHQ/AHQ	0 (0.00)	0 (0.00)	1 (0.33)	0 (0.00)	0 (0.00)		0 (0.00)

### Evaluation of nonsynonymous SNPs of the caprine *PRNP* gene

PolyPhen-2 predicts the possible effect of an amino acid substitution induced by nonsynonymous SNPs on the structure and function of proteins^46^. A total of 10 nonsynonymous SNPs on the caprine *PRNP* gene were assessed by PolyPhen-2. According to the impact degree of nonsynonymous SNPs, 9 nonsynonymous SNPs were predicted into three categories as follows: “benign”: Q101R (0.099) H143R (0.129), N146S (0.024), Q171R (0.035), R211Q (0.447), I218L (0.023); “possibly damaging”: W102G (0.603), R154H (0.934); “probably damaging”: G127S (0.992) (Table 5). We also used PROVEAN to predict the biological impact of the 10 nonsynonymous SNPs of the caprine *PRNP* gene^47^. All 10 nonsynonymous SNPs of the caprine *PRNP* gene were predicted as “neutral” (Table 5).

**Table 5:** Measurement of the effect of amino-acid substitutions of *PRNP* nonsynonymous SNPs in Korean native black goats.

Position	AA_1_	AA_2_	Methods	Score	Prediction
101	Q	R	PolyPhen-2	0.099	Benign
			PROVEAN	−1.046	Neutral
102	W	G	PolyPhen-2	0.603	Possibly damaging
			PROVEAN	−1.554	Neutral
127	G	S	PolyPhen-2	0.992	Probably damaging
			PROVEAN	−1.581	Neutral
143	H	R	PolyPhen-2	0.129	Benign
			PROVEAN	−1.429	Neutral
146	N	S	PolyPhen-2	0.024	Benign
			PROVEAN	−1.057	Neutral
154	R	H	PolyPhen-2	0.934	Possibly damaging
			PROVEAN	−0.261	Neutral
171	Q	R	PolyPhen-2	0.035	Benign
			PROVEAN	−0.619	Neutral
211	R	Q	PolyPhen-2	0.447	Benign
			PROVEAN	−0.177	Neutral
218	I	L	PolyPhen-2	0.023	Benign
			PROVEAN	−0.675	Neutral
240	S	P	PolyPhen-2	Not available	Unknown
			PROVEAN	−0.614	Neutral

**Figure 2:**
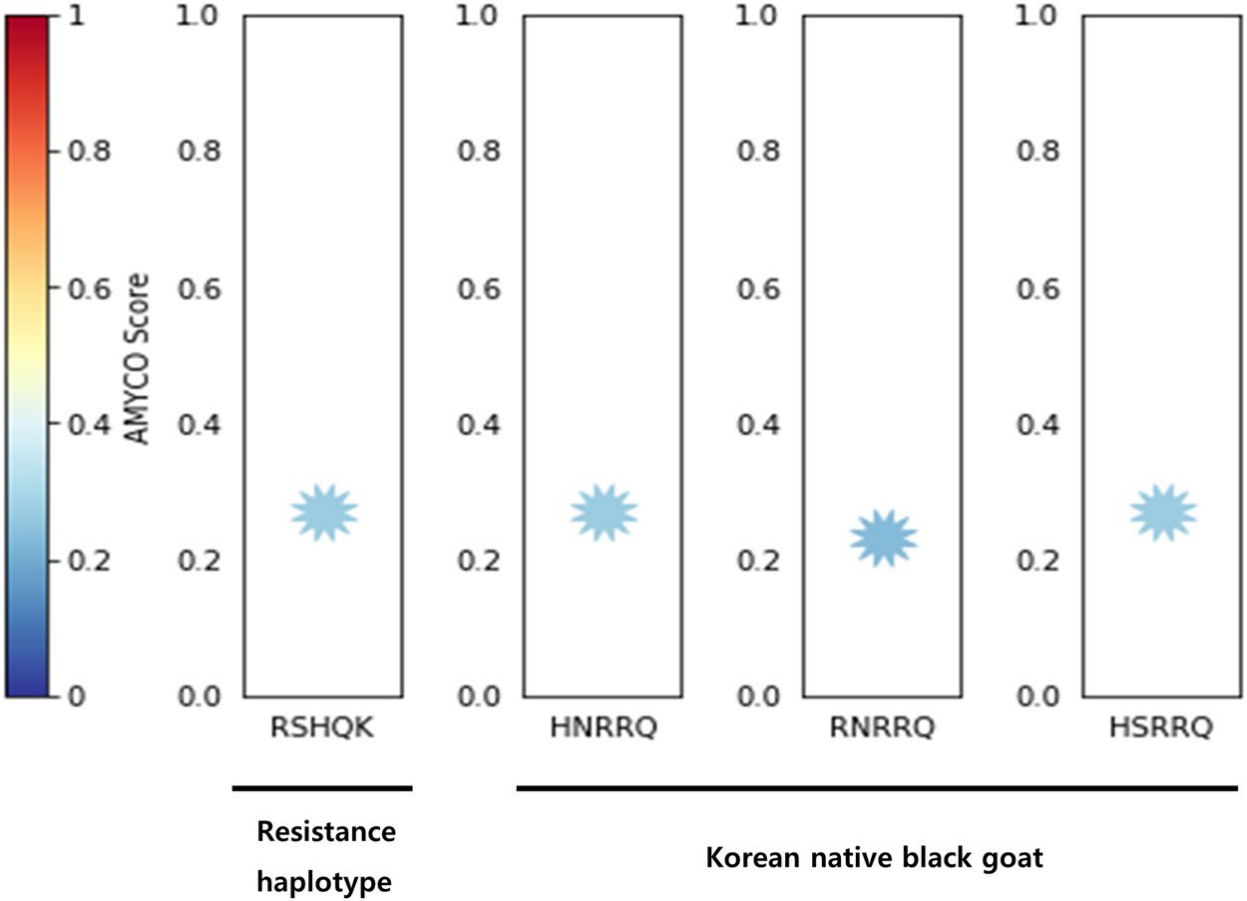
Prediction of amyloid propensity of caprine prion protein according to nonsynonymous SNPs. AMYCO predicted amyloid propensity as values from 0.0 to 1.0. The AMYCO scores < 0.45 and >0.78 indicated low and high aggregation propensities of the protein, respectively. “RSHQK” indicates haplotype of arginine allele at the codon 143, serine allele at the codon 146, histidine allele at the codon 154, glutamine allele at the codon 211 and lysine allele at the codon 222. “HNRRQ” indicates haplotype of histidine allele at the codon 143, asparagine allele at the codon 146, arginine allele at the codon 154, arginine allele at the codon 211 and glutamine allele at the codon 222. “RNRRQ” indicates haplotype of arginine allele at the codon 143, asparagine allele at the codon 146, arginine allele at the codon 154, arginine allele at the codon 211 and glutamine allele at the codon 222. “HSRRQ” indicates haplotype of histidine allele at the codon 143, serine allele at the codon 146, arginine allele at the codon 154, arginine allele at the codon 211 and glutamine allele at the codon 222.

Lastly, we investigated amyloid propensity of goat prion protein according to alleles of nonsynonymous SNPs. Previous studies have been reported that prion protein with alleles of 143R, 146S, 154H, 211Q and 222 K (RSHQK) was highly resistant to conformational change for becoming deleterious form of prion protein. Thus, we analyzed the prion protein based on those alleles. RSHQK haplotype was measured with 0.27 values by AMYCO. In addition, we analyzed the prion protein of Korean native black goats. Amino sequences of prion protein in Korean native black goats were classified to 4 haplotypes (HNRRQ, RNRRQ and HSRRQ) based on alleles of nonsynonymous SNPs. HNRRQ and HSRRQ haplotypes were measured with 0.27 values. RNRRQ haplotype was measured with 0.24 values (Fig. 2).

## Discussion

Polymorphisms of the *PRNP* gene are major genetic determinants of the susceptibility to scrapie in sheep and goats. Although the prion protein in goats shares 99% protein sequence identity with that in sheep, the amino acid residues related to scrapie susceptibility are not identical^22,35^. Previous studies reported that the ovine *PRNP* gene was highly polymorphic and that the distributions of genotype and haplotype frequencies at codons 136, 154, and 171 were strongly related to the susceptibility of the scrapie progression^2,34,48,49^.

We examined the genotype and haplotype distributions of caprine *PRNP* codons 136, 154 and 171 from other countries, including Pakistan, China, Japan and France. As a typical feature, the Val allele of *PRNP* codon 136, which is known to be associated with susceptibility to scrapie in sheep, was not found in goats, and no polymorphisms of codon 136 were found (Table 4). Interestingly, *PRNP* codon 171 polymorphism was found only in Korea and Japan. The major haplotype of the caprine *PRNP* gene was ARQ, and ARR haplotypes were distributed at the lowest level in Japan and Korea. The major genotype ARQ/ARQ was distributed over 97% in Pakistani goats, Chinese goats, Japanese goats, French goats and Korean native black goats. In addition, there was a statistically significant difference in allele distribution between scrapie-affected and healthy goats in France with distributions of 2.39% for the AHQ allele (p < 0.001) and 4.78% for the ARQ/AHQ genotype (p < 0.001). The haplotypes and genotype distributions of French goats were 100% of ARQ and 100% of ARQ/ARQ, respectively, in scrapie-affected animals. The haplotype distribution in Korean native black goats was similar to that in Pakistani goats, Chinese goats and Japanese goats, and the genotype distribution in Korean native black goats was similar to that in Pakistani goats and Chinese goats (Table 4).

Next, we tried to evaluate the potential scrapie susceptibility by comparing distributions of *PRNP* polymorphisms in Korean native black goats with those of scrapie-affected goats. From previous studies, homozygotes of codons 143H, 146N, 154R, 211R and 222Q in the *PRNP* gene are known to be susceptible to goat scrapie^3,33,41,50–52^. On the other hand, heterozygotes of codons H143R, N146S, R154H, R211Q, and Q222K in the *PRNP* gene are associated with a lower risk of developing classical scrapie^3,38,41,52–54^. The *PRNP* codon 168 polymorphism has been reported in Greece, Italy, and Cyprus goats, and 168P homozygotes were associated with scrapie susceptibility^3,37,39,50^. Korean native black goats have 100% of the Ile allele in codon 142, and we did not find genetic polymorphisms. Because the 142I allele was associated with a shorter incubation period in experimentally challenged goats with the TSE isolate^42^, and the I142M heterozygote had a lengthened incubation period after experimental inoculation with BSE and scrapie^42^. In addition, Korean native black goats had similar allele distributions of the *PRNP* gene in codons 154R, 168P, 211R, and 222Q with those of scrapie-affected goats (Fig. 1C–F). In contrast, comparing the allele distributions of *PRNP* polymorphisms between healthy goats and Korean native black goats, codons 143H and 146S of Korean native black goats showed similar distributions to healthy goats (Fig. 1A,B). However, because the caprine *PRNP* gene has too many polymorphisms involved in susceptibility to scrapie, evaluating the susceptibility by comparing the genetic distribution of individual SNPs is difficult. Therefore, we analyzed LD and haplotype among *PRNP* SNPs.

In Korean native black goats, 10 polymorphisms showed strong LD among *PRNP* SNPs (Table 2). We found 4 major haplotypes, QWGHNRQRIP (0.363), QWGRNRQRIP (0.303), QWGHNRQRIS (0.146) and QWGHSRQRIP (0.045), by analyzing the haplotype distributions. (Table 3). Using the haplotype found in this study, a scrapie reagent inoculation test will be helpful for evaluating scrapie susceptibility in Korean native black goats in the future. However, since it cannot be ruled out that the differences of genotype, allele and haplotypes frequencies in the current study were a consequence by the lack of selective pressure due to negligible exposure to scrapie in Korea, additional confirmation studies are needed in the future.

In addition, we predicted the effects of nonsynonymous SNPs on caprine *PRNP* gene by PolyPhen-2 and PROVEAN analyses. In the PolyPhen-2 assay, G127S (0.992) was ‘probably damaging’ and W102G (0.603) and R154H (0.934) were ‘possibly damaging’. Interestingly, all 10 nonsynonymous SNPs of *PRNP* gene were predicted to be ‘neutral’ by PROVEAN. The inconsistencies in the results of PolyPhen-2 and PROVEAN are due to the differences in algorithms that translate the impact on the protein function^46,55^. Because the PolyPhen-2 analysis is based on the structural influence of nonsynonymous SNPs on the protein and the PROVEAN analysis is based on phylogenetic differences among species, we presume that two nonsynonymous SNPs may have an effect on the structure of goat prion protein. Point mutation at the codon 102 of human *PRNP* gene (codon 105 in goat) has been linked to GSS of human familial prion diseases. In addition, polymorphism at the codon 154 of ovine *PRNP* gene (codon 154 in goat) has been associated with the susceptibility of scrapie in sheep. Thus, further investigation of caprine *PRNP* at the codons 102 and 154 is highly desirable in the future.

Next, we evaluated potential scrapie susceptibility according to alleles of nonsynonymous SNPs of caprine *PRNP* gene. Interestingly, the degree of amyloid formation of prion protein according to haplotypes of the *PRNP* gene of the Korean native black goats was lower than or equal to that of prion protein which are known to be resistance to prion diseases. Since scrapie in Korean native black goats has not been reported thus far, this result seems likely to be consistent with the data of AMYCO analysis. Thus, to the best our knowledge Korean native black goat presumed to have resistance to scrapie thus far. However, to verify the nonsynonymous effect of the goat prion protein, it is necessary to perform *in vivo* or *in vitro* experiments using models that contain these two polymorphisms.

Numerous scrapie-related SNPs have been investigated in small ruminants for identifying correlations between the genetic diversity of the *PRNP* gene and scrapie susceptibility in various countries; however, Korean native black goats have not been tested for scrapie susceptibility thus far. Here, we suggest that the multiple *PRNP* alleles of Korean native black goats can predict potential scrapie susceptibility and may contribute to the onset of the disease. In addition, to exclude the probability of quite different frequencies of *PRNP* gene polymorphisms in other regions of Korea, we collected 41 blood samples of Korean native black goats from a slaughter house of another region, which is located in Jecheon-si, Chungcheongbuk-do, Republic of Korea and investigated genotype, allele and haplotype frequencies of caprine *PRNP* gene. The genotype, allele and haplotype frequencies of caprine *PRNP* gene showed similar distributions between Chungcheongbuk-do goats and Jeollanam-do goats (Supplementary Table 1). Since direct transmission of classical scrapie to primates after a 10 years incubation period was recently reported^56^, it is very important to investigate the scrapie susceptibility of Korean native black goats to prevent the possibility of scrapie transmission through the species barrier to humans.

In conclusion, we performed direct sequencing and investigated the genotype and allele distributions of caprine *PRNP* gene polymorphisms in 211 Korean native black goats. We also reported the distributions of 4 major haplotypes and the strong LD among *PRNP* SNPs. Using comparative analysis of the genetic distributions in *PRNP* codons 136, 154 and 171 that are major contributors to the scrapie incidence of sheep, between sheep and Korean native black goats, we confirmed that sheep and Korean native black goats had significant differences in genetic distributions in *PRNP* codons 136, 154 and 171. In addition, we performed a potential scrapie susceptibility test for the first time in Korean native black goats through a comparison of the allele frequencies of previously reported scrapie-associated SNPs. Furthermore, we investigated the damaging impact of nonsynonymous SNPs found in Korean native black goats for the first time using *in silico* analysis tools, PolyPhen-2 and PROVEAN. To the best of our knowledge, we conducted the first *PRNP* genetic study in Korean native black goats.

## Methods

### Ethical statement

All experimental procedures were approved by the Chonbuk National University Institutional Animal Care and Use Committee (IACUC number: CBNU 2017-0076). All experiments using Korean native black goats were performed in accordance with the Korea Experimental Animal Protection Act.

### Blood sample collection and DNA extraction

Blood samples of 211 Korean native black goats were collected from a slaughter house of Hwasun-gun, Jeollanam-do, the Republic of Korea. These samples were provided from 8 farms, which is located in Jeollanam-do. We collected samples 5 times from March 2016 to June 2016. The sample size used in the present study may be enough to identify rare polymorphisms, including below 1% genotype frequency. In addition, the sample size can also represent the total population of Korean native black goats with a 95% confidence level and a confidence interval of 7. Whole blood samples were treated with ethylenediaminetetraacetic acid (EDTA) and were frozen at −80 °C prior to analysis. Genomic DNA was purified from 200 μl frozen blood using the QIAamp DNA Blood Mini Kit (Qiagen, Valencia California, USA) following the instructions from the supplier.

### Polymerase chain reaction (PCR) and DNA sequencing

To amplify the caprine *PRNP* gene, PCR was performed with gene-specific primers as follows: caprine *PRNP*-Forward (5′-ATTTTGCAGAGAAGTCATCATGGTGA-3′) and caprine *PRNP*-Reverse (5′-AACAGGAAGGTTGCCCCTATCCTA-3′). The primers were designed based on the genomic sequence of the caprine *PRNP* gene, which was registered in the GenBank website (Gene ID: EU870890.1). The PCR mixture contained 10 μM of each primer, 2.5 μl of 10 × *Taq* polymerase reaction buffer containing 25 mM of MgCl_2_, 2.5 mM of each dNTP mixture, and 2.5 units of Solg^TM^ *Taq* DNA polymerase (SolGent, Daejeon, Republic of Korea). The PCR was carried out as follows: predenaturation at 95 °C for 2 min, 30 cycles of denaturation at 95 °C for 20 sec, annealing at 58 °C for 40 sec, extension at 72 °C for 1 min, and final extension at 72 °C for 5 min. The purification of PCR products for sequencing analysis was performed with a QIAquick Gel Extraction Kit (Qiagen, Valencia California, USA). The PCR products were directly sequenced by an ABI 3730XL sequencer (Applied Biosystems, Foster City, California, USA).

### Statistical analysis

Analysis of HWE, LD and haplotype distributions of the *PRNP* gene in Korean native black goats were performed using Haploview Version 4.2 (Broad Institute, Cambridge, MA, USA). Differences in genotype, allele and haplotype frequencies of the *PRNP* gene were tested by chi-squire test (χ^2^) or Fisher’s exact test using SAS 9.4 Software (SAS Institute Inc., Cary, NC, USA).

### Evaluation of nonsynonymous SNPs in the caprine prion protein

We evaluated a total of 10 nonsynonymous SNPs of the *PRNP* gene using PolyPhen-2 (http://genetics.bwh.harvard.edu/pph2/) and PROVEAN (http://provean.jcvi.org/index.php). PolyPhen-2 utilizes a naive Bayes classifier to provide independent count (PSIC) scores using information from sequence alignment and protein structural properties^46,47^. PolyPhen-2 predictions are subdivided into three types, “benign”, “probably damaging”, and “possibly damaging”, according to a numerical score ranging from 0.0 to 1.0. PROVEAN calculates the impact score by predicting the functional effect on all classes of protein functions for protein sequence changes such as deletion and multiple substitution as well as single amino acid substitutions^47^. The PROVEAN program predicts the biological impacts of nonsynonymous SNPs, including effects on protein function and structure. Score predictions form PROVEAN have two types of threshold; scores below −2.5 are considered “deleterious”, and scores above −2.5 are considered “neutral”. Amyloid propensity of caprine prion protein according to alleles of *PRNP* SNPs was analyzed by AMYCO (http://bioinf.uab.cat/amyco)^57^. AMYCO is the algorithm used to predict amyloid fibril propensity from amino acid sequences.

## Supplementary information

Supplementary Table 1
