# Supplementary material for: Potential scrapie-associated polymorphisms of the prion protein gene (PRNP) in Korean native black goats
Source: Sci Rep. 2019 Oct 25;9:15293. doi: 10.1038/s41598-019-51621-y (PMC6814802; doi:10.1038/s41598-019-51621-y)
Supplement: Supplementary file 1 — Supplementary Table 1 [file 41598_2019_51621_MOESM1_ESM.docx]

**Potential scrapie-associated polymorphisms of the prion protein gene (*PRNP*) in Korean native black goats**

Seon-Kwan Kim^1, 2, 3^, Yong-Chan Kim^1, 2, 3^, Sae-Young Won^1,2^, Byung-Hoon Jeong^1, 2*^

^1^Korea Zoonosis Research Institute, Chonbuk National University, Iksan, 54531, Republic of Korea

^2^Department of Bioactive Material Sciences and Institute for Molecular Biology and Genetics, Chonbuk National University, Jeonju, 54896, Republic of Korea

***Correspondence:** Byung-Hoon Jeong, Ph.D.

Korea Zoonosis Research Institute, Chonbuk National University,

820-120, Hana-ro, Iksan, Jeonbuk 54531, Republic of Korea.

TEL: 82-63-900-4040, FAX: 82-63-900-4012, E-mail: bhjeong@jbnu.ac.kr

^3^ These authors contributed equally to this work.

**Supplementary Table 1.** Genotype and allele frequencies of eight *PRNP* polymorphisms in Korean native black goats from Jecheon-si area.

| **Polymorphisms** | **Genotype frequency, n (%)** | | | **P-value*** | **Allele frequency, n (%)** | | **HWE**** | **P-value***** |
| --- | --- | --- | --- | --- | --- | --- | --- | --- |
| c.126G>A | GG | GA | AA |  | G | A |  |  |
| 42P | 23 (56.10) | 16 (39.02) | 2 (4.88) | 0.7823 | 62 (75.61) | 20 (24.39) | 0.7100 | 0.5631 |
|  |  |  |  |  |  |  |  |  |
| c.302A>G | AA | AG | GG |  | A | G |  |  |
| Q101R | 41 (100.00) | 0 (0.00) | 0 (0.00) | 1.0 | 82 (100.00) | 0 (0.00) | - | 1.0 |
|  |  |  |  |  |  |  |  |  |
| c.304T>G | TT | TG | GG |  | T | G |  |  |
| W102G | 39 (95.12) | 2 (4.88) | 0 (0.00) | 1.0 | 80 (97.56) | 2 (2.44) | 0.8728 | 1.0 |
|  |  |  |  |  |  |  |  |  |
| c.379G>A | GG | GA | AA |  | G | A |  |  |
| G127S | 40 (97.56) | 1 (2.44) | 0 (0) | 1.0 | 81 (98.78) | 1 (1.22) | 0.9370 | 1.0 |
|  |  |  |  |  |  |  |  |  |
| c.414T>C | TT | TC | CC |  | T | C |  |  |
| 138S | 23 (56.10) | 16 (39.02) | 2 (4.88) | 0.8291 | 62 (75.61) | 20 (24.39) | 0.7100 | 0.8892 |
|  |  |  |  |  |  |  |  |  |
| c.426A>G | AA | AG | GG |  | A | G |  |  |
| I142M | 41 (100) | 0 (0.00) | 0 (0.00) | - | 82 (100.00) | 0 (0.00) | - | - |
|  |  |  |  |  |  |  |  |  |
| c.428A>G | AA | AG | GG |  | A | G |  |  |
| H143R | 26 (63.41) | 11 (26.83) | 4 (9.76) | 0.1945 | 63 (76.83) | 19 (23.17) | 0.1146 | 0.1783 |
|  |  |  |  |  |  |  |  |  |
| c.437A>G | AA | AG | GG |  | A | G |  |  |
| N146S | 35 (85.37) | 5 (12.20) | 1 (2.44) | 0.1704 | 75 (91.46) | 7 (8.54) | 0.1607 | 0.1666 |
|  |  |  |  |  |  |  |  |  |
| c.461G>A | GG | GA | AA |  | G | A |  |  |
| R154H | 41 (100) | 0 (0.00) | 0 (0.00) | 1.0 | 82 (100.00) | 0 (0.00) | - | 1.0 |
|  |  |  |  |  |  |  |  |  |
| c.503C>A | CC | CA | AA |  | C | A |  |  |
| P168Q | 41 (100) | 0 (0.00) | 0 (0.00) | - | 82 (100.00) | 0 (0.00) | - | - |
|  |  |  |  |  |  |  |  |  |
| c.512A>G | AA | AG | GG |  | A | G |  |  |
| Q171R | 41 (100) | 0 (0.00) | 0 (0.00) | 1.0 | 82 (100.00) | 0 (0.00) | - | 1.0 |
|  |  |  |  |  |  |  |  |  |
| c.632G>A | GG | GA | AA |  | G | A |  |  |
| R211Q | 41 (100) | 0 (0.00) | 0 (0.00) | 1.0 | 82 (100.00) | 0 (0.00) | - | 1.0 |
|  |  |  |  |  |  |  |  |  |
| c.652A>C | AA | AC | CC |  | A | C |  |  |
| I218L | 40 (97.56) | 1 (2.44) | 0 (0) | 0.5108 | 81 (98.78) | 1 (1.22) | 0.9370 | 0.6348 |
|  |  |  |  |  |  |  |  |  |
| c.664C>A | CC | CA | AA |  | C | A |  |  |
| Q222K | 41 (100) | 0 (0.00) | 0 (0.00) | - | 82 (100.00) | 0 (0.00) | - | - |
|  |  |  |  |  |  |  |  |  |
| c.718C>T | CC | CT | TT |  | C | T |  |  |
| P240S | 27 (65.85) | 14 (34.15) | 0 (0) | 0.5589 | 68 (82.93) | 14 (17.07) | 0.1874 | 0.5221 |

**P-value*:** Difference of genotype distribution of the caprine *PRNP* polymorphisms between Hwasun-gun and Jecheon-si Korean native black goats

**HWE**:** Hardy–Weinberg equilibrium

**P-value***:** Difference of allele distribution of the caprine *PRNP* polymorphisms between Hwasun-gun and Jecheon-si Korean native black goats
